# Quad Shot—A Tertiary Hospital Experience With a Palliative Radiotherapy Regimen for Head and Neck Cancer

**DOI:** 10.1002/hed.28202

**Published:** 2025-06-02

**Authors:** Eirini Nikolaidou, Tilman Bostel, Arnulf Mayer, Daniel Wollschläger, Heinz Schmidberger, Justus Kaufmann

**Affiliations:** ^1^ Department of Radiation Oncology University Medical Center Mainz Mainz Germany; ^2^ Department for Radiation Oncology Charité‐Universitätsmedizin Berlin Berlin Germany; ^3^ Radiologisches Institut Dr. von Essen Koblenz Germany; ^4^ Department of Radiation Oncology Peter MacCallum Cancer Center Melbourne Australia; ^5^ Institute for Medical Biostatistics, Epidemiology and Informatics, University Medical Center Mainz Mainz Germany

**Keywords:** accelerated radiotherapy, head and neck squamous cell carcinoma, hypofractionation, palliative care, symptom control

## Abstract

**Background:**

Head and neck cancer patients often present with locally advanced disease. For some patients, palliative treatment schedules focused on symptom control are often preferred. Perhaps the most widely used of these is the so‐called Quad Shot (QS) regimen.

**Methods:**

We evaluated 51 patients with head and neck squamous cell carcinomas treated with QS at University Medical Centre Mainz between 2017 and 2024 for response, survival, and toxicity.

**Results:**

Median overall survival was 5.9 (95% CI: 4.3–10.5) months. Thirty‐nine patients could be evaluated for response; out of these, 16 patients had an objective response, and 21 patients had stable disease. Only two cases of toxicity Grade 3 or higher were observed and are most likely attributable to other reasons.

**Conclusion:**

QS was well tolerated and showed a meaningful response in most patients. Using real‐world data from a highly palliative patient cohort, we discuss several considerations for QS prescription.

## 1 Background

Head and neck squamous cell carcinoma (HNSCC) is one of the most common malignancies worldwide [1]. Due to underlying risk factors and associated comorbidities, patients often present with locally advanced disease, which would only be curable using treatments associated with significant morbidity [2, 3]. Some patients are not amenable to radical treatment with curative intent due to several reasons, such as poor performance status, age, or severe comorbidities. In other patients, tumors might have metastasized or present too advanced to undergo surgery or radiotherapy without substantial risk of severe complications [4].

For these patients, palliative treatment regimens focused on local and symptomatic control are preferred [5, 6]. Such regimens aim to keep adverse treatment effects to a minimum while hoping to achieve durable relief of symptoms. The so‐called “Quad Shot” (QS) regimen is perhaps the most well‐known palliative treatment regimen for this situation.

QS is a cyclical hypofractionated radiotherapy regimen consisting of twice‐daily radiation with 3.5 Gy on two consecutive days for a total of 14 Gy repeated every 4 weeks for up to three cycles and a maximum dose of 42 Gy [7, 8]. Several other treatment strategies such as the “0‐7‐21” regimen or the “Christie scheme” can also be used in this scenario [9, 10, 11, 12, 13, 14].

All of these regimens use hypofractionated radiotherapy as a means to reduce overall treatment time (OTT). In the case of QS, combination with immunotherapy has been shown to increase progression‐free survival (PFS) considerably [8].

We adopted QS as a palliative standard of care in our clinic in 2017. In this study, we analyze volumetric changes of the tumor and factors associated with improved outcomes after QS in a real‐world data set from a tertiary center in Germany.

## 2 Methods

### 2.1 Patient Data

In this single‐center retrospective study, all 51 patients with HNSCC treated with QS at our institution between 2017 and June 2024 were included. This retrospective study was approved by our local ethics committee (Ethics Vote No: 2022‐16 800‐retrospective). Due to the purely retrospective nature of this study based solely on the clinical data recorded at regular clinical visits, no informed consent had to be obtained.

### 2.2 Data Collection

We performed an extensive review of clinical records and digital images of all patients. The collected data included demographics, clinical notes, comorbidities, medication, course of the disease, radiotherapy courses, lab results, and outcome data. Symptoms were assessed mainly by review of clinical notes. In addition, if not documented, the Karnofsky Performance Score (KPS) was based on clinical notes. To assess response to treatment, tumor volumes were contoured and calculated in the radiotherapy planning software. For this, contours were generated at two time points: first in every slice of the planning CT, and second in either the last planning CT available prior to termination of RT or in the CT at the 3‐month follow‐up after completion of the third QS cycle. ACE‐27 Score [15, 16] was calculated from clinical notes and physician's letters. HNSCC was ignored for the calculation of the ACE‐27 Score. The primary outcome of this study was overall survival (OS) after the end of RT. Secondary outcomes included PFS and tumor response. Response was evaluated according to RECIST criteria using available imaging after the last applied QS cycle. This could be normal staging CT or MRI or planning CT for the next cycle of QS, in case further therapy was not deemed beneficial after evaluating response. Patients who received only one cycle of QS without further follow‐up were excluded from the response assessment. Tumor volume was calculated by contouring the visible tumor and pathological lymph nodes in all slices.

### 2.3 Radiation Treatment

In all patients, RT was planned based on planning CT and performed by linear accelerators using intensity‐modulated radiotherapy (IMRT) or volumetric arc therapy (VMAT) combined with image guidance. Patients were immobilized in the supine position using standard thermoplastic masks. New planning CT was performed prior to every QS cycle. Patients were evaluated by the treating physicians during the planning CT for adverse events and response. In case of progressive disease or severe deterioration of performance status, RT was terminated.

### 2.4 Statistical Analysis

Data analysis was performed using R Version 4.2.3 [17]. Two‐tailed Kruskal–Wallis test, Wilcoxon rank‐sum test, and chi‐square test were used to compare differences between groups. Pairwise post hoc Wilcoxon tests after the Kruskal–Wallis test were carried out using Bonferroni correction for multiple testing. Correlation was assessed using Spearman's rho. Survival data was plotted using the Kaplan–Meier method. Due to long treatment times, OS time was computed both from the start of RT and from the last day of RT. We report OS after RT for survival comparisons between groups where immortal time bias due to substantially differing lengths of RT might bias results. Potential clinical factors influencing OS were subjected to univariate analysis using a log‐rank test. We report restricted mean OS when median OS was not reached. Differences were considered statistically significant for *p* < 0.05.

## 3 Results

### 3.1 Patient and Tumor Characteristics

In this study, 51 patients with a median age of 73 years (interquartile range [IQR]: 66–82 years) who had received QS for HNSCC were included. Most patients had poor performance status of ECOG 3 or 4 (63%, *n* = 32) and severe comorbidities as described by an ACE‐27 score of 2 or higher (65%, *n* = 33). Fourteen patients (27%) had received prior RT to the neck, while QS was chosen as a de‐escalation treatment for six patients (12%) who deteriorated while receiving RT with curative intent. Patients who received reirradiation had received a median dose of 63.9 Gy (IQR: 55.5–70 Gy) prior to QS treatment.

In 19 patients, HPV status could not be assessed. Where accessible, most patients were HPV‐negative (72%, *n* = 23/32). The most common primary tumor locations were the oral cavity (43%, *n* = 22) and oropharynx (21.5%, *n* = 11), and most patients had Stage IV disease (80%, *n* = 41). The mean tumor volume was 112 ccm (range: 7–1235 ccm) (Table 1).

**TABLE 1:** Patient characteristics.

Characteristics	*N* = 51^a^
Age	73 (66–82)
Elderly	
Elderly	29 (57%)
Non‐elderly	22 (43%)
Sex	
Male	36 (71%)
Female	15 (29%)
Performance score	
ECOG 1	9 (18%)
ECOG 2	10 (20%)
ECOG 3	25 (49%)
ECOG 4	7 (14%)
Comorbidities (ACE‐27‐Score)	
0	10 (20%)
1	8 (16%)
2	19 (37%)
3	14 (27%)
Primary cancer location	
Oral cavity	22 (43%)
Oropharynx	11 (22%)
Hypopharynx	4 (7.8%)
Nasopharynx	3 (5.9%)
Nasal cancer	1 (2.0%)
Major salivary glands	6 (12%)
Laryngeal cancer	4 (7.8%)
Prior radiotherapy	
No prior radiotherapy	37 (73%)
Prior radiotherapy	14 (27%)
Concurrent systemic therapy	
No systemic therapy	38 (75%)
Chemotherapy	1 (2.0%)
Chemo‐/immunotherapy	8 (16%)
Other	2 (3.9%)
Chemo‐/cetuximab	2 (3.9%)
Systemic treatment after radiotherapy	
No systemic therapy	38 (75%)
Chemotherapy	3 (5.9%)
Chemo‐/immunotherapy	7 (14%)
Other	2 (3.9%)
Chemo‐/cetuximab	1 (2.0%)

Abbreviation: ECOG, Eastern Cooperative Oncology Group.

^a^ *n* (%).

### 3.2 Treatment Characteristics

Three RT cycles could be administered in 41% of patients (*n* = 21), while 33% of patients received only one QS cycle (*n* = 17). Only 5 out of 14 patients with prior RT to the neck (36%) completed the initially planned RT. Out of those five patients, only two patients received three cycles. Concurrent systemic therapy was given in 25% of patients (*n* = 13) with some form of immunotherapy being given in 61.5% of these patients (*n* = 8/13). RT was discontinued early in 47% of patients (*n* = 24). The most common reasons for early termination of RT were deterioration of patients' performance status (37.5%; 9/24), patient death (33%; 8/24), and a combination of insufficient response or progression and prior RT (16.67%; 4/24). Two patients had progressive disease between cycles, and one patient had progressive dementia and refused further treatment. More details are given in Table 2.

**TABLE 2:** Tumor characteristics.

Characteristics	*N* = 51^a^
T stage	
0	4 (7.8%)
1	0 (0%)
2	7 (14%)
3	11 (22%)
4	29 (57%)
N stage	
0	16 (31%)
1	31 (61%)
2	1 (2.0%)
3	3 (5.9%)
UICC stage	
1	1 (2.0%)
2	5 (9.8%)
3	4 (7.8%)
4	41 (80%)
PD‐L1 TPS	
< 1	8 (28%)
1–5	8 (28%)
5–20	7 (24%)
20+	6 (21%)
Unknown	22
Treatment setting	
Primary	29 (57%)
Recurrence	22 (43%)
HPV status	
HPV‐negative	23 (72%)
HPV‐positive	9 (28%)
Concurrent systemic therapy	
No systemic therapy	38 (75%)
Chemotherapy	1 (2.0%)
Chemo‐/immunotherapy	8 (16%)
Other	2 (3.9%)
Chemo‐/cetuximab	2 (3.9%)

Abbreviations: HPV, human papilloma virus; PD‐L1TPS, PD‐L1 tumor positivity score.

^a^ *n* (%).

### 3.3 Survival Outcomes

Median OS and PFS from the start of RT were 5.87 months (95% confidence interval [CI]: 4.3–10.5 months) and 2.7 months (95% CI: 1.2–5.53 months) respectively. One‐year and 2‐year OS from the start of RT were 23% (95% CI: 13.6%–41%) and 11% (95% CI: 4%–29%) respectively. The median follow‐up time was 21.2 months (95% CI: 12.4 months–NA). Patients had significantly better OS after RT if they received three cycles of QS (1.2 months vs. 8 months, *p* < 0.001).

**FIGURE 1:**
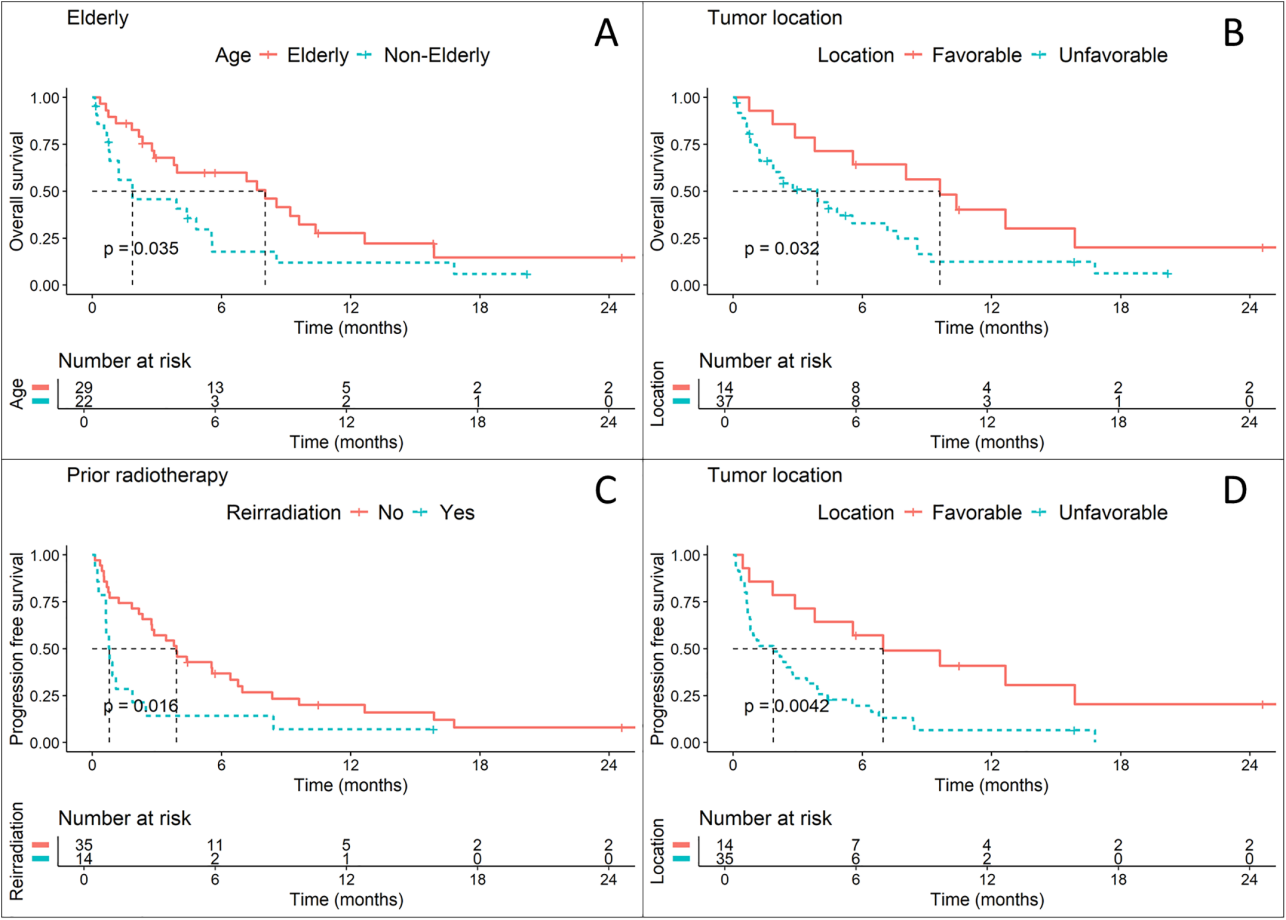
(A and B) Overall survival in patients stratified by age (A) and tumor location (B). Patients were considered elderly if they were at least 70 years old. Tumor location was defined as favorable in patients with p16‐positive oropharyngeal carcinomas, major salivary gland carcinomas, and nasopharyngeal carcinomas. (C and D) Progression‐free survival in patients stratified by treatment situation (C) and tumor location (D). [Color figure can be viewed at wileyonlinelibrary.com]

Factors associated with improved OS were application of systemic therapy after QS (median OS: 9.6 vs. 3.8 months; *p* < 0.01) and—unexpectedly—age over 70 (median OS: 8 vs. 1.9 months; *p* < 0.05) (Figure 1A). Concerning tumor location, nasopharyngeal carcinoma and HPV‐associated oropharyngeal carcinoma were expectedly associated with better OS. However, to our surprise, major salivary gland cancer showed survival rates similar to HPV‐associated oropharyngeal and nasopharyngeal carcinomas. Grouped, these three “favorable” locations were significantly associated with better OS and PFS (median OS: 9.6 vs. 3.9 months; *p* < 0.05) (Figure 1B).

Improved PFS was also associated with the application of systemic therapy after QS (median PFS: 8.4 vs. 1.8 months; *p* < 0.01) and a favorable location (median PFS: 7 vs. 1.9 months; *p* < 0.01) (Figure 1D). Age above 70 showed a non‐significant trend for improved OS (median PFS: 3.1 vs. 0.9 months; *p* = 0.06). Using QS as a reirradiation regimen (Figure 1C) was associated with poor PFS (median PFS: 3.9 vs. 0.8 months; *p* < 0.05), although this did not translate to a significant difference in OS.

Early discontinuation of QS due to deteriorating patient status or progression was unsurprisingly associated with poorer OS (median OS: 8.6 vs. 1.8 months; *p* < 0.001) and PFS (median PFS: 6.4 vs. 0.8 months; *p* < 0.001).

Nine patients (18%) lived longer than 1 year after starting QS. Details of these patients are compared to the remaining patients in Table 3.

**TABLE 3:** Characteristics of patients living for at least 1 year after Quad Shot treatment and those having shorter survival.

Factor		Survival for at least 1 year	Survival for less than 1 year	*p*
Age	Mean (SD)	78.9 (11.4)	71.8 (10.9)	0.086
ECOG	ECOG 0	0 (0.0)	0 (0.0)	0.702
	ECOG 1	1 (11.1)	8 (19.0)	
	ECOG 2	3 (33.3)	7 (16.7)	
	ECOG 3	4 (44.4)	21 (50.0)	
	ECOG 4	1 (11.1)	6 (14.3)	
Simultaneous systemic treatment	No systemic therapy	4 (44.4)	34 (81.0)	0.057
	Chemotherapy	0 (0.0)	1 (2.4)	
	Chemo‐/immunotherapy	4 (44.4)	4 (9.5)	
	Other	1 (11.1)	1 (2.4)	
	Chemo‐/cetuximab	0 (0.0)	2 (4.8)	
Comorbidities	ACE 0–1	5 (55.6)	13 (31.0)	0.309
	ACE 2–3	4 (44.4)	29 (69.0)	
Primary or recurrence	Primary	4 (44.4)	25 (59.5)	0.647
	Recurrence	5 (55.6)	17 (40.5)	
Prior RT	No prior RT	7 (77.8)	30 (71.4)	1.000
	Prior RT	2 (22.2)	12 (28.6)	
Prior RT dose	Mean (SD)	58.0 (39.4)	47.2 (22.4)	0.495
Tumor site	Favorable	**6 (66.7)**	**8 (19.0)**	**0.013**
	Unfavorable	**3 (33.3)**	**34 (81.0)**	
UICC stage	UICC I–III	2 (22.2)	8 (19.0)	1.000
	UICC IV	7 (77.8)	34 (81.0)	
T stage	T0–T2	1 (11.1)	10 (23.8)	0.694
	T3–T4	8 (88.9)	32 (76.2)	

*Note:* Significant differences were only observed for the tumor site.

### 3.4 Tumor Response

**FIGURE 2:**
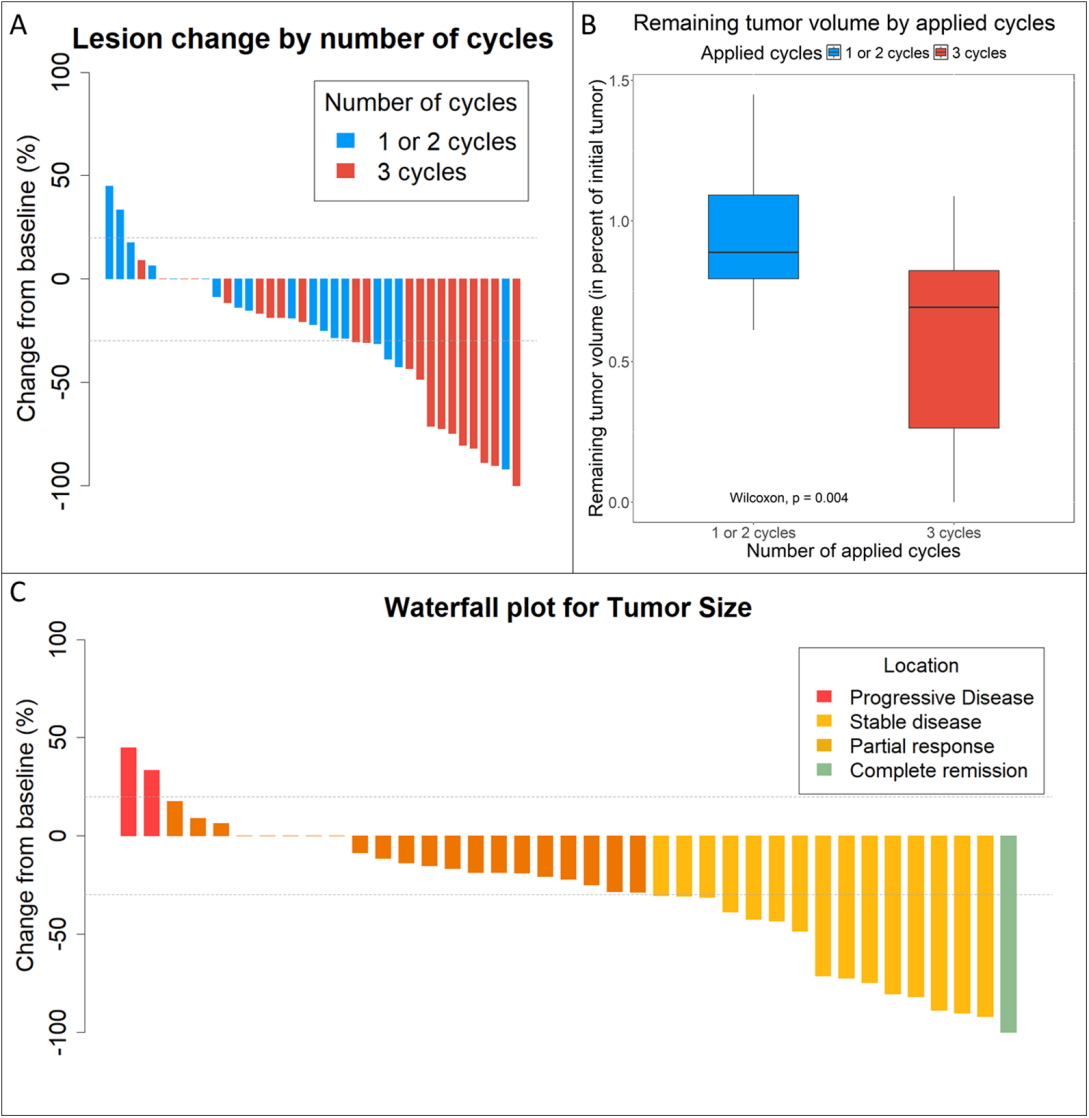
(A) Waterfall plot depicting changes in tumor size from baseline for all patients who had imaging after radiotherapy available. Each case is color coded by number of applied cycles. Gray dotted lines show cutoffs for progressive disease and partial remission according to RECIST. (B) Differences in remaining tumor volume, stratified by number of applied cycles. (C) Waterfall plot depicting changes in tumor size from baseline. Each case is colored by tumor response according to RECIST. [Color figure can be viewed at wileyonlinelibrary.com]

In patients who did not receive all three cycles, the median tumor volume after therapy was 89% of the initial tumor volume (IQR: 79%–109% of initial tumor volume). In patients who received the full dose of QS, median tumor volume after therapy was 69% (IQR: 26%–82% of initial tumor volume). Wilcoxon rank‐sum test showed a significant difference (*p* < 0.01) in remaining tumor volume between patients who received one or two QS cycles compared with the full three cycles (Figure 2).

Thirty‐nine patients could be assessed for tumor response. Of those, 15 patients had at least a partial response, with 1 patient having a complete response. Twenty patients had stable disease, and four patients had progressive disease. Disease progression occurred mostly after the first cycle (*n* = 3) and in one case after the second cycle. Tumor volume was reduced in median by 72% (IQR: 42%–84% volume reduction) in patients who had at least partial response.

### 3.5 Toxicity

QS was well tolerated by the whole patient collective, despite poor performance status, serious comorbidities, or old age. We only observed two serious adverse events Grade 3 or higher. One patient receiving concomitant cetuximab developed Grade 4 dermatitis, and one patient developed Stevens–Johnson syndrome after antibiotic therapy for a superinfected lymphatic fistula about 4 weeks after the first cycle of QS. This condition was confirmed in histopathology. One patient who had received prior radiotherapy died due to tumor erosion of the carotid artery 4 days after the second cycle of QS. Although possible, we think carotid blowout to be unlikely, as the maximum point dose to the carotid artery was below 100 Gy, and the time between radiotherapy and the event of bleeding was very short. We therefore counted this as tumor progression, rather than Grade 5 toxicity. Low‐grade toxicity was more common and mainly limited to xerostomia (18% of patients, *n* = 9), radiodermatitis (6% of patients, *n* = 3), and mucositis (10% of patients, *n* = 5). Fifty‐nine percent of patients had no adverse events documented (Table 4).

**TABLE 4:** Distribution of toxicity.

	Toxicity grade (CTCAE version 05)			Total
	1	2	4	
Toxicity				
Loss of taste	1	1	0	2
Mucilage	0	2	0	2
Mucositis	3	2	0	5
Oedema	2	0	0	2
Radiodermatitis	2	0	1	3
Xerostomia	9	0	0	9
Total	17	5	1	23

*Note:* Grade 4 Radiodermatitis occurred in a patient who received concurrent cetuximab and developed Stevens–Johnson Syndrome.

## 4 Discussion

In this paper, we describe the clinical results of patients treated with the QS regimen in our clinic. If considered feasible, dose‐reduced normofractionated or mildly hypofractionated regimens, for example, 50 Gy in 20–25 fractions, are preferably prescribed for palliative treatment in our clinic.

QS in our clinic is generally prescribed as a last resort either for patients with a poor performance status (ECOG 3 or higher), frail elderly patients who decline other therapies, patients with very bulky tumors not amenable to more intense treatment, or in case of poor prognosis. In case of especially poor performance status with an expected prognosis of less than 3 months, only one cycle of QS is prescribed prior to transferring patients to hospice care. As such, survival in our cohort was expectedly poor, with the main goal often being symptom control rather than prolongation of life. In this regard, we feel that achieving at least a stable disease in nearly 90% of patients who were evaluable is to the benefit of the treated patients. Even if we would consider all patients without follow‐up to have had progressive disease, stable disease would have still been achieved in nearly 70% of patients. This is especially meaningful as toxicity was negligible even in very frail patients.

Our results are in line with other studies, evaluating QS in cohorts of patients with HNSCC, often with a somewhat better prognosis [7, 8].

We think that several important lessons can be learned from this real‐world analysis:

First, several unexpected factors were associated with better OS or PFS. Major salivary gland carcinomas, often considered less radiosensitive, showed a good response to hypofractionated RT [18, 19, 20]. Surprisingly, elderly patients showed better survival. This is most likely due to a selection bias, as the willingness to deescalate therapy in lower tumor stages might be more common in elderly patients, whereas younger patients more likely will receive high‐dose conventional fractionation unless their general condition is very poor. Both the treating physician and the patient probably introduced this bias.

Second, completion of QS after prior RT to the neck was low and most patients had minimal PFS of less than a month after QS. For this reason, we will probably not consider QS as a reirradiation regimen anymore.

Third, in patients who are deemed comparatively fit, other palliative RT regimens, such as the Christie scheme (50 Gy in 16 fractions) or the “0‐7‐21” regimen (24 Gy in 3 fractions on Days 0, 7, and 21), have shown promising results and either improved OS or higher response rates [9, 11]. Toxicity of these regimens, however, was also increased in comparison to QS. Still, some patients might be willing to accept higher toxicity for improved tumor control chances. We feel that clinicians should not focus on one palliative standard across all patients, but rather several RT regimens should be discussed and considered in accordance with the patient's individual goals of care.

Finally, QS still induced good response rates with negligible toxicity. Treatment time is short and can be performed both on an inpatient and outpatient basis. Occasionally, patients present with large and debilitating carcinomas at initial diagnosis. As their overall prognosis and clinical condition are not conducive to lengthy interventions, these patients can be transitioned to hospice care without palliative local treatment in favor of the best supportive care. Due to the short treatment time of QS, time to admission to hospice care is not delayed considerably. Instead of palliative cancer treatment, QS should rather be regarded as a measure of best supportive care in these patients.

Our study is limited due to its retrospective nature. Patient's death dates were obtained by the local cancer registry. While this allows for rigorous survival analysis, toxicity was only assessed by our department before every QS cycle or by other caring physicians in the case of further follow‐up. Although it is in line with other studies in which toxicity and quality of life were evaluated prospectively, our report on treatment‐associated acute toxicity is limited. Patients who received systemic therapy after QS had improved OS and PFS, which we would rather consider to be a result of performance status and immortal time bias. Although this may be attributable to low numbers of patients being deemed fit enough to receive systemic treatment, simultaneous systemic therapy was also unable to demonstrate a significant association with improved OS or PFS.

In conclusion, we feel that QS is a useful highly palliative RT regimen for very frail patients, patients who want to stabilize their current quality of life while avoiding treatment‐associated toxicity, or patients already planned for hospice or best supportive care. However, palliative RT should not be a “one size fits all” approach and treatment regimens have to be chosen on an individual basis.

## Conflicts of Interest

The authors declare no conflicts of interest.

## Data Availability

The data that support the findings of this study are not publicly available due to privacy restrictions according to the German data safety laws.
